# Antibiotic Stewardship Ward Rounds and a Dedicated Prescription Chart Reduce Antibiotic Consumption and Pharmacy Costs without Affecting Inpatient Mortality or Re-Admission Rates

**DOI:** 10.1371/journal.pone.0079747

**Published:** 2013-12-09

**Authors:** Tom H. Boyles, Andrew Whitelaw, Colleen Bamford, Mischka Moodley, Kim Bonorchis, Vida Morris, Naazneen Rawoot, Vanishree Naicker, Irena Lusakiewicz, John Black, David Stead, Maia Lesosky, Peter Raubenheimer, Sipho Dlamini, Marc Mendelson

**Affiliations:** 1 Division of Infectious Diseases and HIV Medicine, Department of Medicine, Groote Schuur Hospital, University of Cape Town, Cape Town, South Africa; 2 National Health Laboratory Service, Groote Schuur Hospital, Cape Town, South Africa; 3 Division of Medical Microbiology, University of Cape Town, Cape Town, South Africa; 4 Division of Medical Microbiology, University of Stellenbosch, Cape Town, South Africa; 5 Quality Assurance, Groote Schuur Hospital, Cape Town, South Africa; 6 Pharmacy, Groote Schuur Hospital, Cape Town, South Africa; 7 Department of General Medicine, University of Cape Town, Cape Town, South Africa; 8 Division of General Internal Medicine, Groote Schuur Hospital, University of Cape Town, Cape Town, South Africa; California Department of Public Health, United States of America

## Abstract

**Background:**

Antibiotic consumption is a major driver of bacterial resistance. To address the increasing burden of multi-drug resistant bacterial infections, antibiotic stewardship programmes are promoted worldwide to rationalize antibiotic prescribing and conserve remaining antibiotics. Few studies have been reported from developing countries and none from Africa that report on an intervention based approach with outcomes that include morbidity and mortality.

**Methods:**

An antibiotic prescription chart and weekly antibiotic stewardship ward round was introduced into two medical wards of an academic teaching hospital in South Africa between January-December 2012. Electronic pharmacy records were used to collect the volume and cost of antibiotics used, the patient database was analysed to determine inpatient mortality and 30-day re-admission rates, and laboratory records to determine use of infection-related tests. Outcomes were compared to a control period, January-December 2011.

**Results:**

During the intervention period, 475.8 defined daily doses were prescribed per 1000 inpatient days compared to 592.0 defined daily doses/1000 inpatient days during the control period. This represents a 19.6% decrease in volume with a cost reduction of 35% of the pharmacy’s antibiotic budget. There was a concomitant increase in laboratory tests driven by requests for procalcitonin. There was no difference in inpatient mortality or 30-day readmission rate during the control and intervention periods.

**Conclusions:**

Introduction of antibiotic stewardship ward rounds and a dedicated prescription chart in a developing country setting can achieve reduction in antibiotic consumption without harm to patients. Increased laboratory costs should be anticipated when introducing an antibiotic stewardship program.

## Introduction

Global concern exists that we are now facing a post-antibiotic era [1], caused by decades of injudicious antibiotic use driving the emergence of multi-drug resistant (MDR) Gram-negative bacterial infections [2]. The incidence of resistant Gram-positive bacterial infections such as methicillin-resistant *Staphylococcus aureus* (MRSA) and vancomycin-resistant enterococci (VRE) is also rising internationally and in South Africa [3,4]. Whilst the development of novel antibiotics for Gram-positive infections has kept pace with emergence of resistant bacteria, the antibiotic pipeline for new drugs active against Gram-negative bacteria has dried up, with none expected on the market for the next 10-15 years. The alarming increase in rates of extended-spectrum beta-lactamase (ESBL)-producing Gram-negative bacteria being reported from South African hospitals [4] and countrywide outbreaks of Carbapenem-Resistant Enterobacteriaceae (CRE) [5,6] are a cause for grave concern. Compounding the problem is the lack of infection prevention control capacity in South Africa [7], which is unable to contain the spread of resistant bacterial infections. The current situation is a direct threat to patient safety. 

Acquisition of drug-resistant hospital-acquired infection (HAI) increases morbidity, length of hospital stay and mortality [8]. Antibiotic use drives resistance [9,10] and therefore any unnecessary antibiotic use, irrespective of class adds to selection pressure for resistant bacteria. A point prevalence study of antibiotic prescriptions in Intensive Care Units across South Africa documented patients receiving up to 10 antimicrobials simultaneously [11]. Moreover, for public and private intensive care unit patients, inappropriate antibiotics were prescribed in 43.5% and 73% respectively, and for an inappropriate duration in 53.2% and 81.7% respectively. Widespread abuse of antibiotic prescribing is also occurring in primary care, often fuelled by patient expectations and poor education surrounding the potential harm of antibiotics [12].

Antibiotic stewardship programmes (ASP) aim to combat antibiotic misuse. Antibiotic stewardship is a multifaceted, multidisciplinary team approach to optimise antibiotic prescribing. The approach includes the formulation of policies, use of treatment guidelines, surveillance data, education resources, targeted interventions and audit. Interventions to reduce excessive inpatient antibiotic prescribing reduce resistance, HAI, and improve clinical outcome [13]. We introduced a 2-component intervention aimed at reducing overall antibiotic consumption without increasing morbidity and mortality at a busy South African academic teaching hospital in Cape Town, South Africa. 

## Methods

### Ethics Statement

The University of Cape Town Faculty of Health Sciences Human Research Ethics Committee (HREC) approved the study. Approval was given for the use of oral informed consent, as registries and databases where anonymized standard of care data is captured do not require informed consent by the HREC. However for this clinical audit, oral consent for the use of anonymized routine data from drug charts and laboratory records was requested from each patient, but was not documented in the patient’s file.

Groote Schuur Hospital (GSH), the 945-bedded tertiary academic teaching hospital of the University of Cape Town, offers medical, surgical, obstetric, psychiatric and emergency care to Cape Town’s metro west population. Consultant-led antibiotic prescribing is the norm, with restriction of certain antibiotics such as carbapenems, colistin, tigecycline and vancomycin, which require release by a microbiologist or infectious diseases physician. Web-based educational material regarding spectra of antibiotic coverage and an interactive case-based antibiotic tutorial are freely available on the University’s intranet. All doctors at GSH have access to the Western Cape Academic Hospitals Antibiotic Recommendations [14], which are updated annually.

Beginning on 22^nd^ November 2011, we piloted the introduction of a dedicated antibiotic prescription chart and a weekly antibiotic stewardship ward round in 2 general medical wards comprising 32 beds each. These wards admit general medical patients via the emergency unit, with patients managed by 5 consultant-led teams who admit patients over a 24-hour period. Each ward has 4 single rooms for isolation of patients requiring airborne or contact precautions.

### Antibiotic Prescription Chart

**Figure 1:**
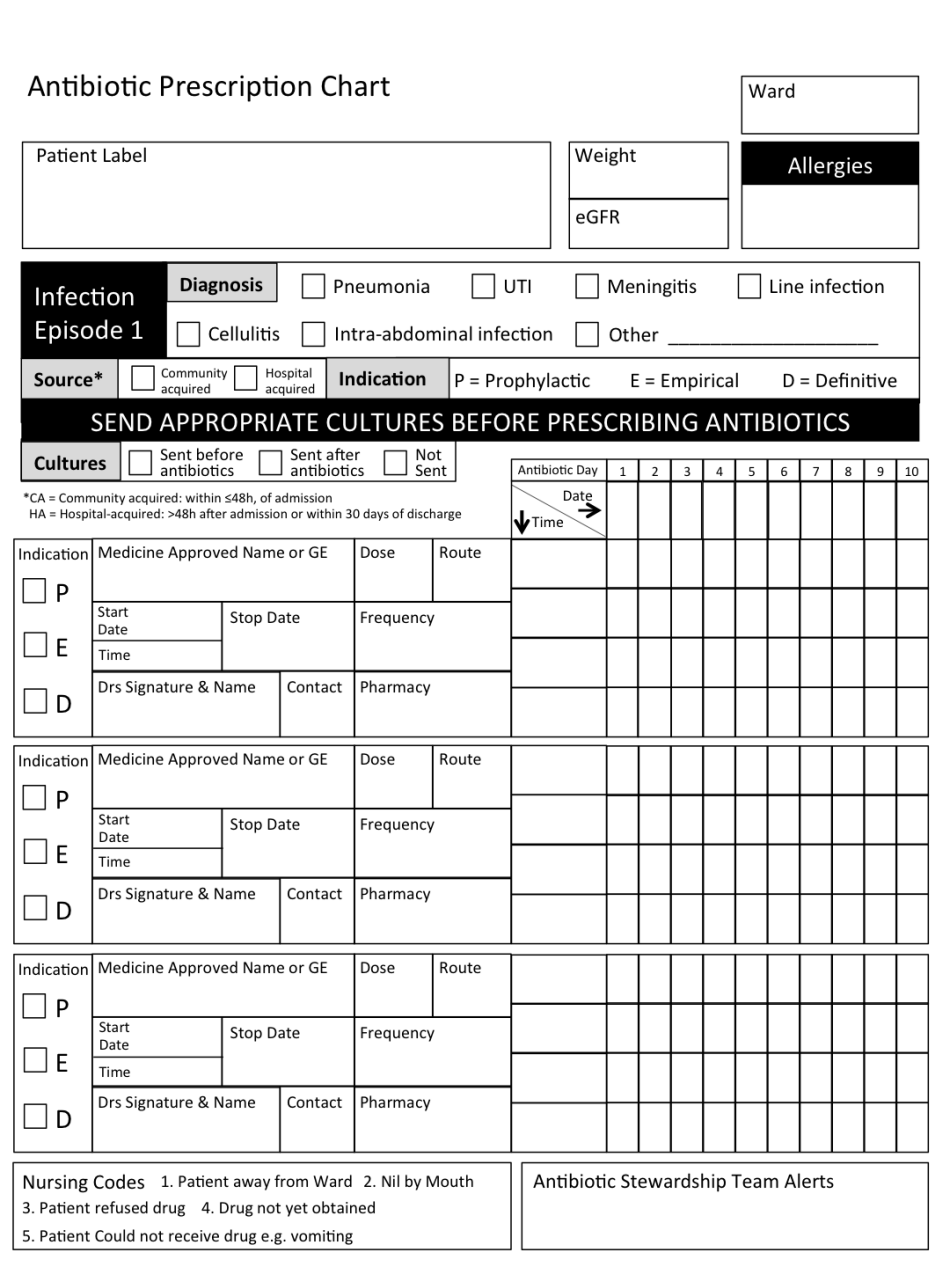
Antibiotic Prescription Chart [25].

An antibiotic prescription chart (Figure 1) was designed to focus antibiotic prescribing on distinct infection episodes. A patient admitted with an infection requiring antibiotics or an inpatient admitted with a non-infectious condition who subsequently developed a HAI was prescribed antibiotics for ‘Infection episode 1’. Any subsequent, distinct infection would be prescribed antibiotics under ‘Infection episode 2’. The categorization necessitated the prescribing doctor to define the following parameters for each infection episode; the indication for antibiotics, whether antibiotics were being prescribed on prophylactic (P), empiric (E) or definitive (D; antibiotic prescription based on microbiological culture and sensitivity) grounds, whether the infection was community-acquired (symptoms starting in the community or within 48 hours of admission) or hospital-acquired (symptoms starting >48 hours after hospital admission), and whether appropriate specimens had been sent for laboratory culture before or after antibiotics had started. Each chart permitted 3 separate infection episodes to be documented as well as single dose prescribing. The last page of the chart contained information for prescribers detailing types of laboratory specimens to send, pharmacological information on drug interactions, antibacterial spectrum and therapeutic monitoring as well as guidance on duration of therapy, based on the Western Cape Academic Hospitals Antibiotic Recommendations 2012. The prescription chart was used solely for antibiotics. Antifungals and antiviral drugs were prescribed on the normal hospital drug chart, as were long-term prophylactic antibiotics such as cotrimoxazole in HIV-infected patients. Prophylactic antibiotics that were prescribed on the antibiotic prescription chart related to surgical prophylaxis. Antibiotic prescription charts were placed in the emergency unit (EU) and on both medical wards. Medical teams were instructed accordingly. 

### Antibiotic Stewardship Ward Rounds

Antibiotic stewardship (AS) ward rounds were conducted on a weekly basis alternating between the 2 medical wards. An infectious diseases specialist, consultant microbiologist, infection prevention control nurse, and ward pharmacist comprised the core AS team. A ward nurse and medical registrars supervising patient care joined each round. Every patient on the ward was reviewed, a pertinent history given and antibiotic prescribing reviewed. Each case was then discussed, an action plan agreed upon, and education around AS imparted. The types of intervention recommended by the AS team are shown in Table 1. In addition, the necessity for indwelling urinary catheters and intravenous cannulae was reviewed and removal undertaken whenever possible. We also ensured that the correct infection prevention control signage was employed. Data on antibiotic use was collected during the AS ward rounds.

**Table 1:** Types of intervention recommended by the antibiotic stewardship team.

Stop antibiotics when there was no indication for use or if multiple antibiotics with overlapping spectrum were prescribed
Start antibiotics when indicated
Change in dose, including adjustment for renal dysfunction and weight
Change in duration
Change in frequency of administration
Change in route of administration, most commonly switching from parental to oral or nasogastric
De-escalation of empiric broad spectrum antibiotic to narrow-spectrum antibiotic based on the antibiogram
Escalation of empiric narrow spectrum to empiric broad spectrum antibiotic based on clinical deterioration of the patient and laboratory indicators, when no bacteria had been identified
Removal of indwelling urinary catheter or intravenous cannulae
Adoption of appropriate infection prevention and control practice including isolation of the patient, use of appropriate signs and personal protective equipment for health care workers

### Audit of antibiotic prescription chart use

To determine compliance with chart use, point prevalence audits of all patients on both wards were conducted once weekly for four weeks during August / September 2012. These were separate from the AS ward rounds. The use of a chart when prescribing antibiotics was recorded; in addition the rate of completion of 12 separate indicators on charts was also assessed.

### Antibiotic use

Electronic pharmacy dispensing records were used to calculate consumption of each antibiotic, with differentiation of oral and parenteral formulations of the same antibiotic. Antibiotic use during the intervention period (1^st^ January - 31^st^ December 2012) was compared to that during the control period (1^st^ January 2011 - 31^st^ December 2011). Consumption of antibiotics was converted into defined daily dosages (DDDs) according to the World Health Organization standard [15].

During each AS ward round, a point prevalence survey was performed to determine the number of patients currently receiving antibiotics and the number who had received antibiotics earlier in their admission but were not currently receiving them.

### Laboratory tests

The number and costs of blood cultures, full blood counts, white blood cell count differential, C-reactive protein (CRP) and procalcitonin (PCT) requests during the control and intervention periods was calculated from the National Health Laboratory Service database. 

### Patient data

Data on in-patient mortality and re-admission to Groote Schuur Hospital within 30-days of discharge was obtained from the hospital electronic admissions database (Clinicom). The Chi-Square test was used to assess statistically significant differences in in-patient mortality and re-admission rates. 

## Results

### Antibiotic prescription chart audit

The records of 136 patients who had been prescribed antibiotics were audited over the 4-week period and an antibiotic prescription chart was used in 130 (96%). In total there were 263 unfilled fields, median 2 per chart (range 0-6). The fields most likely to be omitted from charts were weight (45% of charts), estimated glomerular filtration rate (36%), allergies (32%), and the ward that the patient was on (26%).

### Point prevalence surveys of antibiotic usage

**Figure 2:**
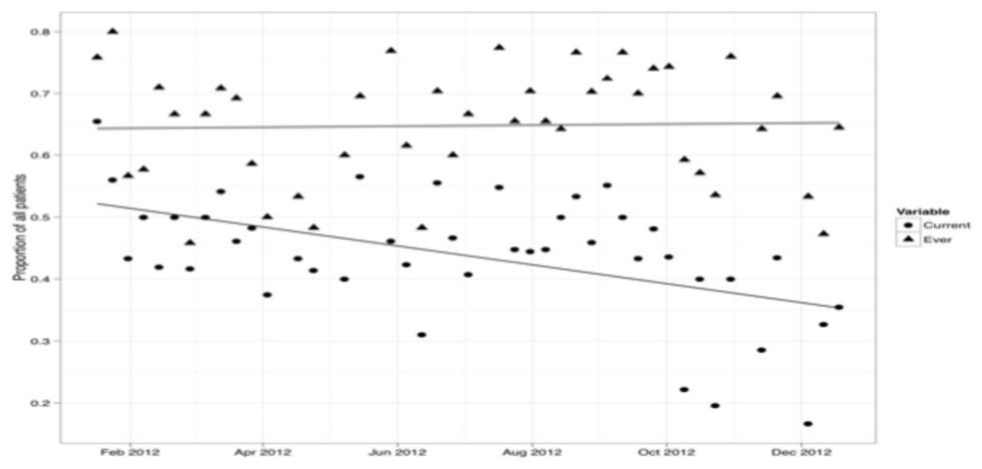
Results of 43-point prevalence surveys undertaken during the intervention period (January–December 2012), showing proportion of patients during their current admission who ever received antibiotics (diamonds) and the proportion currently receiving antibiotics (crosses).

During the intervention period, 43-point prevalence surveys of antibiotic use were conducted, covering 1249 patient episodes. The proportion of patients who had ever received antibiotics (currently or previously on the same admission) remained constant during the intervention period at approximately 65%. In contrast, there was a gradual decline in the proportion of patients who were currently receiving antibiotics (Figure 2).

### Change in antibiotic consumption and cost

**Figure 3:**
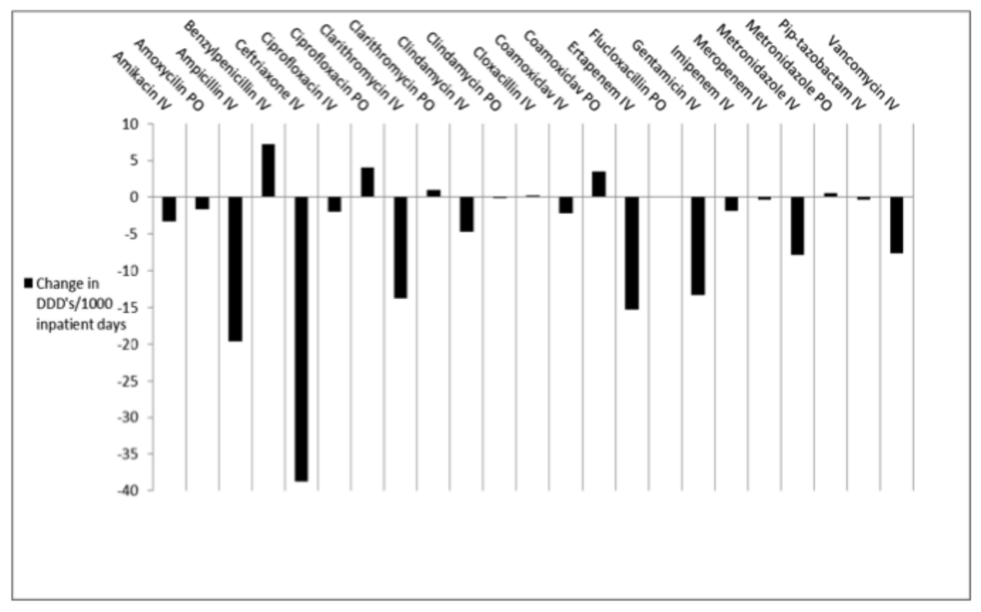
Change in Defined Daily Doses of antibiotics per 1000 inpatient days, between control period (January–December 2011) and intervention period (January–December 2012) on 2 general medicine wards.

Total consumption of antibiotics, expressed in DDDs/ 1000 inpatient days on the 2 medical wards was 592.0 and 475.8 during the control and intervention periods respectively, representing a 19.6% reduction in antibiotic consumption. Figure 3 shows the change in use of individual antibiotics. Most notably, there was a reduction of high-volume use antibiotics such as parenteral ceftriaxone (-38.7 DDDs/1000 patient days), ampicillin (-19.6) and ertapenem (-15.3). There were small increases in the use of oral co-amoxiclav, ciprofloxacin and clarithromycin with corresponding decreases in use of the intravenous formulations of the same drugs. The total cost of antibiotics during the control period was R1 068 325 compared to R694 705 during the intervention period representing a cost saving of R373 620 (35%).

### Laboratory tests

Changes in the use of laboratory tests between control and intervention periods are summarized in Table 2. There was an increase in the use of most tests, notably a doubling in use of CRP and fivefold increase in the use of PCT. Total costs for the laboratory tests were R463 580 during the control period and R608 232 during the intervention period representing an increase of R144 652 (31%). The total increase in cost of laboratory tests was driven in the main by the increase in PCT use, whose percentage of the total costs of laboratory tests of 5% in 2011, increased to 22% of the total in 2012.

**Table 2:** Number of laboratory tests performed during the control and intervention phase.

	Control (2011)	Intervention (2012)	Percentage Change
Full blood count	5 645	5 853	3.7
White blood count	364	446	22.5
C-Reactive Protein	310	619	100
Procalcitonin	61	367	502
Blood Culture	1 924	1 896	-1.5

### Removal of urinary catheters and peripheral intravenous cannulae

On average, 33 patients were seen on each AS ward round. The recorded average number of urinary catheters and peripheral intravenous cannulae removed from patients during each ward round was 1.9 (5.7%) and 2.1 (6.3%) respectively. 

### Inpatient mortality and readmission rates

During the control period, 2427 patients were admitted to the 2 medical wards with a mean age of 48 years (standard deviation [SD] 18 years), compared to 2517 patients, mean age of 50 years (SD 18 years) during the intervention period. A total of 311 (12.8%) inpatients died and 226 (9.3%; 95% CI 8.2-10.5) were re-admitted to the hospital within 30 days in the control period compared to 315 (12.5%) inpatient deaths and 213 (8.5%, 95% CI 7.4-9.6%) patients re-admitted during the intervention period.

## Discussion

The key finding of this study is that the introduction of AS ward rounds and a dedicated antibiotic chart resulted in 19.6% reduction in the total volume of antibiotic use and a cost reduction of 35% of the pharmacy budget in 2 general medical wards in a developing country setting. Importantly, the intervention was not associated with ‘collateral damage’ i.e. unpredicted adverse effects, in terms of inpatient mortality or 30-day readmission rate to the hospital. However, a concomitant increase in laboratory costs was incurred, mainly due to an increase in the use of procalcitonin tests. 

This study adds to the pertinent body of knowledge by demonstrating the utility of an AS intervention in a developing country such as South Africa. In the latest Cochrane Systematic Review of interventions to improve antibiotic prescribing practices for hospital inpatients, none of the 89 studies included in the review were from Africa and only 5 were from developing countries i.e. Brazil, Colombia and Thailand [13].

The effect of the AS programme on the profile of Gram-negative bacterial antibiotic resistance in our hospital is being studied longitudinally. However, in the face of increasing countrywide antibiotic resistance and transfer of patients into our hospital already colonized or infected with MDR infections, demonstrating a reduction due to the intervention will be challenging.

We witnessed a steady decline in the number of patients still on antibiotics when seen on the weekly ward rounds due to early discontinuation of the antibiotics initially started in the EU or ward. The commonest reasons for this were inadequate indication for antibiotics or an inappropriate duration of antibiotics charted by the admitting physician. For example, prior to the intervention, it was commonplace for ceftriaxone to be prescribed for 2 weeks for all patients admitted with community-acquired pneumonia or sepsis of unknown cause. Following the introduction of AS ward rounds, this duration decreased to a standard of 5 days, except for infections such as meningitis and others requiring prolonged duration of antibiotics. We also demonstrated an increase in initial use of oral over parenteral antibiotics, and an increase in earlier parenteral to oral switch. This was particularly evident for ciprofloxacin, clarithromycin and co-amoxiclav.

Ideally, one would use a stepwise introduction of a single intervention to judge benefit. On this occasion, we opted for a dual intervention strategy as the antibiotic prescription chart also acted as an audit tool. Although we cannot disregard the possibility that the chart itself may have contributed to the reduced antibiotic use, we hypothesize that the major factor effecting change was the AS ward round. Ward rounds were held once weekly, yet the early stopping of antibiotics commonly occurred prior to the weekly ward round, indicating change in prescribing practice. Furthermore, the AS ward round was used to train and transfer skills, particularly in complex cases where there is often a question of equipoise. A number of studies from different healthcare settings in the United States support our hypothesis, having shown reduction in antibiotic prescribing and cost following input by trained specialists in the decision making process [16-19].

Antibiotic restriction reduces antibiotic use and cost [20]. However, limiting one antibiotic or antibiotic class commonly leads to increased prescribing of other antibiotics that are not restricted. This ‘squeezed balloon effect’ ensures that total volume of antibiotic use may not change. Furthermore there is no evidence that antibiotic restriction leads to long-term reduction in resistance. In our own setting, an antibiotic restriction programme has been in place for many years at Groote Schuur Hospital, whereas at the neighbouring Tygerberg Hospital, a tertiary academic teaching hospital of University of Stellenbosch, a very similar antibiotic resistance profile is present despite the hospital having abandoned antibiotic restriction 7 years ago. 

A number of studies have documented benefit from introduction of dedicated antibiotic prescription charts. Closest to our own chart design, Durbin et al reported reduction in inappropriate prophylactic antibiotic use and mean duration of prophylactic antibiotics, as well as an increase in appropriate antibiotic prescribing in urology patients from 38% to 89%, following an introduction of a new chart [21]. Similarly, a study that introduced a dedicated prescription chart in an 800-bedded hospital requiring an indication and defined duration to be charted, reduced overall antibiotic use by 30% and the overall hospital pharmacy budget by 2% over 25 months [22].

Laboratory costs associated with the introduction of the AS programme at GSH increased. A gatekeeping strategy was in place to limit the use of CRP. However, no such strategy was in place for PCT at the start of our intervention. The majority of the excess costs were attributed to inappropriate PCT use, often with repeated or serial measurements. Although serial measurement of PCT has been shown to be of benefit in guiding when to stop antibiotics, particularly in the intensive care unit setting [23,24], in a resource-poor setting such as the South African public health system, this is not a strategy that has been adopted as part of hospital practice. Furthermore, >2 PCT estimations were occasionally requested by doctors in an attempt to define whether to start antibiotics. This was also deemed inappropriate practice. Guidelines for the use of PCT were introduced resulting in a reduction of tests performed in the last quarter of the intervention period. This illustrates the importance of taking into account how an AS programme can impact on hospital finances over and above just the antibiotic costs. Although savings in antibiotic costs were diminished by the increase in laboratory costs, we did not factor in savings from reduced use of equipment to deliver intravenous antibiotics, reduction in length of stay of patients having switched earlier from intravenous to oral antibiotics and other cost savings. Therefore, the overall reduction in cost of R228, 968 represents an underestimate.

There are several limitations to our study. As highlighted, the use of a 2-part intervention makes it difficult to dissect out the contribution made by the chart or the ward rounds. Secondly, the AS team described in this intervention is not reproducible outside of a tertiary, or rarely a secondary level hospital in South Africa or many developing countries. Infectious diseases specialists and microbiologists are usually domiciled at tertiary centres, and although they perform outreach and support to primary level facilities, the same composition of AS team is unlikely to be replicated. However, the principle of stewardship and a team constructed around key role players such as pharmacists and physicians with appropriate training could be introduced at any health care facility. One of the objectives of the South African Antibiotic Stewardship Programme [25] is to develop training programmes for pharmacists and non-specialist physicians to enable AS in all healthcare facilities.

We did not record baseline demographic patient information or severity of illness indicators, which leaves us unable to accurately assess differences in the inpatient populations during the control and intervention years. However, we did not witness any gross differences in patient profile during the 2 years, both of which had mild influenza seasons. Our results to date do not allow us to determine what effect the intervention has had on antibiotic resistance rates in our hospital. Although antibiotic consumption is a major driver of resistance, introduction of resistant organisms from other hospitals will confound the issue. Our intervention on 2 medical wards, does not allow us to generalize in terms of its applicability in different clinical settings. However, preliminary results from a similar ongoing intervention in the general surgical wards at our hospital, is also showing a reduction in total volume of antibiotic use and pharmacy cost (data not shown). 

Future research questions will be aimed at addressing the issues of reproducibility of the intervention in different healthcare settings, change in resistance patterns, and optimizing individual patient outcomes through AS. Reducing hang time (the time from writing up the prescription to entry of antibiotic into the patient), optimizing antibiotic dose and rational therapeutic drug monitoring are important research questions to address. 

We face an uncertain future of MDR Gram-negative bacterial infections. The rise in CRE and its characterization has highlighted the broad resistance genes these bacteria carry and the ability of most antibiotics to select out these resistant organisms. Hence, interventions to reduce antibiotic consumption are urgently needed to limit the emergence of resistant organisms and must be introduced alongside strengthening of basic infection control practices, such as hand washing. Antibiotic prescription charts and more importantly the rollout of AS ward round activity by AS teams in every healthcare institution will be critical in reducing the volume of antibiotic use and slowing the march towards a post-antibiotic era. 
